# Nutrition prescription for climbing athletes on rock or artificial surfaces: are we guessing?

**DOI:** 10.3389/fspor.2026.1799533

**Published:** 2026-07-31

**Authors:** Lanae Joubert, Marisa Michael, Camryn Nugent, Anna Chmielewska

**Affiliations:** 1 School of Health and Human Performance, Northern Michigan University, Marquette, MI, United States; 2 Real Nutrition, LLC, West Linn, OR, United States; 3 Department of Dietetics and Bromatology, Wroclaw Medical University, Wroclaw, Poland

**Keywords:** rock climber, sport lead, bouldering, dietary intake, energy, macronutrients, micronutrients, eating

## Abstract

Evidence-based sports nutrition recommendations for well-researched sports (i.e., running, cycling, soccer) are predominantly developed from accumulated clinical trial research. In this type of research athletes undergo diet manipulation and donate tissues in order to help inform best nutrition practices for performance success. However, these experiments are often expensive and logistically complex, with a small number of participants. Thus, observing common dietary practices of athletes are a common first step before employing clinical trial methods. In the sport of climbing (on rock and/or on artificial surfaces) no clinical trials have examined diet manipulation so evidence-based sports nutrition guidance has not yet been developed. Although, observational studies involving the dietary intake of climbers have started to accumulate. This minireview aimed to critique the methodology shortcomings of the observational dietary studies of climbers. Results unveiled 16 observational studies, most with meager methods to assess the dietary intakes of 658 climbers (43% female; ages 10–42y) with varying climbing disciplines and abilities. The majority of studies employed recall methodology with very few assessing dietary intake with weighed food records. Additionally, total energy expenditure was estimated in all studies with only three specifically measuring resting metabolic/energy rate/expenditure with indirect calorimetry. These findings emphasize the need for future nutrition climbing research to focus on gold standard methodology for dietary assessment and energy expenditure simultaneously during different training phase periods. Based on our findings, we propose recommendations to guide future research and practice.

## Introduction

Evaluating food consumption and the dietary strategies of humans is a challenging task. It often starts with observations of food intake, analyses of energy, macro- and micronutrients, followed by comparisons with a set of known dietary standards to determine strengths and weaknesses. Finally, a nutrition prescription is set by a nutrition professional intended to moderate the dietary flaws.

Beyond comprehensive dietary assessments, integrating training load parameters and periodized adaptations is fundamental to sports nutrition. While aligning nutritional intake with sport-specific demands optimizes performance (1, 2), experimental interventions examining such dietary manipulations in climbers remain absent from the literature.

Identifying nutritional factors that enhance performance, mitigate injury risk, and preserve long-term health is essential for extending athletic longevity. Although preliminary nutritional recommendations (3, 4) and concerns regarding energy deficiency (5) have been documented, no experimental trials have yet established definitive best practices for this population. Consequently, synthesizing existing cross-sectional observational data is a necessary preliminary step toward establishing evidence-based guidelines.

This minireview aimed to critique the methods of observational studies examining the diets of climbers on rock and artificial surfaces. Ultimately, robust observational data will provide the requisite foundation for future experimental trials and targeted nutritional guidance for competitive climbers.

## Methodology

For this minireview, a structured comprehensive search was performed on PubMed, Scholar, CINAHL full text and ScienceDirect on March 30, 2025. These databases were selected based the researchers access and their depth and breadth of nutrition or diet-related publications. The Preferred Reporting Items for Systematic reviews and Meta-Analyses extension for Scoping Reviews guidelines were followed. Predetermined search terms focused on the diet of climbers on natural rock and artificial surfaces and are reported in Supplementary Table S1.

Studies were considered if they included an analysis of nutrients from food consumption lasting a minimum of 24 h and at a minimum had assessed dietary energy, carbohydrate, protein, and fat. Furthermore, studies were required to be conducted in humans who had at least 6 months of climbing experience on natural outdoor rock or artificial surfaces (i.e., indoor gym), and written in the English language. Studies were excluded if they were not peer-reviewed, did not report dietary intake methods or reveal the food/nutrient database or software employed.

Thirteen published papers met these criteria. Additionally, two abstracts (6, 7) and a graduate student's master thesis (8) data were included only in tables for comparisons.

The primary focus was to critique the methodology of observational studies that assessed the dietary intake (energy, macros, micros, fluids) of climbers. However, the data was organized by characteristics of the study participants, dietary consumption of energy and macronutrients, followed by micronutrients, fluids, then other important sports nutrition considerations. The discussion section focuses on methodology shortcomings and evidence quality, gaps in the literature, and suggestions for future investigations.

### Characteristics of subjects

A total of 658 subjects provided dietary data reported in 16 different studies (6–21) conducted in 9 countries. There were more males (*n* = 373) represented than females (*n* = 285), with the majority of studies including climbers between the ages of 19–42 years of age. Only two studies included adolescent climbers (13, 17). Climbing ability of all adult subjects ranged from 10 to 26 on the International Rock Climbing Research Association (IRCRA) ability scale (22), with the majority between 17 and 20 or an advanced level 3 ability when reported. Very few were classified as high elite or competitive climbers. Only three studies specifically stated their subjects were competitive climbing athletes (12, 13, 17), including the only two in youth athletes (13, 17). Female adult body weight ranged from 52 ± 4.1 to 58 ± 8.9 kg and 65 ± 4.4 to 82 ± 10.0 kg for males in published studies. See Tables 1, 2 for characteristics and macronutrient data and Supplementary Table S2 and S3 to compare micronutrient dietary data.

**Table 1:** Male energy and macronutrient data.

IRCRA Climb Ability	Age (y)	BW (kg)	Dietary Energy	Dietary Carbohydrate	Dietary Protein	Dietary Fat	Dietary Intake Method	Energy Expenditure Method	Country (reference)
(n)			(kcals)	(g/d^c^ or g/kg BW^d^)	(g/d^c^ or g/kg BW^d^)	(g/d^c^ or g/kg BW^d^)	(software)	(derived estimate or instrument)	
24.7 ± 1.9 (20)	26.3 ± 3.7	65.5 ± 4.9	2652	55% energy 5.5 ± 1.2^d^	15% energy 1.5 ± 0.3^d^	27.1 ± 7.3% energy 1.2 ± 0.4^d^	7 d weighed food (ProdiExpert Plus)	NR	Germany (7)
28-36 (15)	28.7 ± 4.1	67.4 ± 4.7	2667	54% energy 393.0^c^	13% energy 87.2^c^	29% energy 86.0^c^	5 d weighed food (Prodi Expert)	NR	Germany (9)
14.5 (2)	21	82.5	3556 ± 477	54% energy 4.7 ± 1.8^d^	54% energy 1.0 ± 0.4^d^	54% energy 2.1 ± 1.9^d^	29 d weighed food (DietAnalysisPlus)	Estimated RMR (Mifflin) Estimated TEE (PA diary, METs)	Canada (10)
		81.7	*2627.8* ± *1286*	51% energy *4.3* *±* *1.7*^d^	51% energy *1.1* *±* *0.6*^d^	51% energy *1.3* *±* *1.0*^d^			
NR (15)	22.4 ± 3.2^a^	64.9 ± 8.9^a^	2125 ± 472	270.0 ± 76.0^c^	91.0 ± 23.0^c^	*77.4* ^c^	3 d food recall (software not reported)	NR	USA (6)
23 ± 1 (13)	NR	68.8 ± 4.4	2337 ± 517	49% energy *286.3*^c^ 4.2 ± 1.1^d^	*109.3* *±* *0.*9^c^ 1.6 ± 0.4^d^	32% energy *84.1*^c^ 1.2 ± 0.4^d^	7 d food diary (Polish Institute of Food & Nutrition Tables)	NR	Poland (11)
9-16 (30)	26.2 ± 4.8	71.1 ± 6.2	2543 ± 566	311.1 ± 80.0^c^	111.0 ± 35.0^c^	101.0 ± 37.0^c^	3 d food diary (FoodProcessor; ESHA)	Estimated BMR (Tanita BCA 545)	Poland (12)
17-29 (35)	27.8 ± 6.1	72.4 ± 7.5	2720 ± 742	333.0 ± 88.0^c^	126.0 ± 50.0^c^	108.0 ± 47.0^c^			
19.8 ± 3.8 (13)	14.8 ± 1.5	61.1 ± 12.4	1963 ± 581	*262.7* *±* *19.8*^c^ 4.3 ± 1.6^d^	*110.0* *±* *12.4*^c^ 1.8 ± 1.0^d^	*71.0* *±* *22.0*^c^	3 d food recall (Nutritics)	Estimated RMR (Nutritics; Schofield)	USA (13)
23.4 (20)	29.1 ± 5.4	69.4 ± 5.8	2270 ± 562	251.7 ± 61.1^c^	109.5 ± 34.8^c^	90.9 ± 29.6^c^	3 d weighed food (Nutritics)	NR	UK (14)
19.1 ± 3.9 (34)	34.3 ± 8.0	69.4 ± 6.9	*2123* ± *648*	*42% energy* *221.3* ^c^	*17% energy* *92.5* ^c^	*36% energy* *85.5* ^c^	3 d food diary (DIAL® Alce Ingeniería)	Estimated BMR (WHO) Estimated TEE (PA diary, METs)	Spain (15)
16.9 ± 3.3 (29)	30.2 ± 5.7	73.0 ± 7.9	2217 ± 429	243.0 ± 61.3^c^ 3.4 ± 0.9^d^	96.3 ± 18.9^c^ 1.3 ± 0.3^d^	86.4 ± 22.7^c^ 1.2 ± 0.3^d^	3 d food diary (Food Processor; ESHA)	Measured RMR (indirect calorimetry; Cosmed Fitmate)	Poland (16)
21.1 ± 2.2 (13)	15.5 ± 1.6	56.4 ± 11.0	1880 ± 470	*232.2* *±* *11.1*^c^ *4.3* *±* *1.3*^a^^,^^d^	*70.2* *±* *0.5*^c^ *1.3* *±* *0.4*^a^^,^^d^	32 ± 5% energy^a^ *63.2* *±* *2.4*^a^^,^^c^	3 d weighed food (OPEN)	Estimated EEE (PA diary, METs)	Slovenia (17)
17.7 ± 4.1 (66)	30.9 ± 6.1	72.4 ± 7.7	2260 ± 508	245.6 ± 67.0^c^ 3.75^d^	100.3 ± 28.9^c^ 1.38^d^	87.5 ± 30.3^c^ 1.18^d^	3 d food diary (Food Processor; ESHA)	Measured RMR (indirect calorimetry; Cosmed Fitmate) Estimated EEE (questionnaire & PA diary, METs)	Poland (18)
23.0 ± 3.4 (14)	26.6 ± 6.4	69.7 ± 8.2	2480 ± 572	261.0 ± 70^c^ 3.8 ± 1.2^d^	109.7 ± 39.1^c^ 1.6 ± 0.6^d^	109.6 ± 39.6^c^ 1.6 ± 0.5^d^	4 d food diary (Nutritics)	Estimated RMR (Cunningham) Estimated EEE (accelerometry, HR, & PA diary)	Ireland (19)
20.0 ± 4.6 (22)	32 ± 10	69.8 ± 5.6	2177 ± 505	210.6 ± 73.8	115.0 ± 27.3	96.4 ± 24.6	3 d food diary (DietPro)	Estimated EEE (questionnaire, METs)	Spain (20)
NR (32)	27.8 ± 4.7	65.0 ± 10.0	1633 (528)^b^	183.8 (78.8)^c^ 2.9 (1.6)^d^	71.6 (26.9)^c^ 1.2 (0.5)^d^	81.2 ± 23.6^c^ 1.2 (0.4)^d^	7 d diet history multi-pass method (Nutritionist Pro)	Measured REE (indirect calorimetry; Cortex MetaMax) Estimated TEE (questionnaire & PA recall, METs)	Malaysia (21)

Reported dietary data assumed from food and beverage only, excluding dietary supplements, in male subjects (*n* = 373) with at least 6 months of climbing experience. Absolute energy and macronutrients are presented as mean ± SD unless otherwise noted.

n, Number of subjects; y, year; kg, kilogram; kcal, kilocalorie; g/d, grams per day; BW, body weight; NR, not reported; BMR, basal metabolic rate; RMR, resting metabolic rate; REE, resting energy expenditure; PA, physical activity; TEE, total energy expenditure; EEE, exercise energy expenditure; HR, heart rate; METs, metabolic equivalents. *Italic font:* estimated from reported data.

a Averaged with female data in same study.

b Data reported as median (interquartile).

c g/d.

d g/kg BW.

**Table 2:** Female energy and macronutrient data.

IRCRA Climb Ability	Age (y)	BW (kg)	Dietary Energy	Dietary Carbohydrate	Dietary Protein	Dietary Fat	Dietary Intake Method	Energy Expenditure Method	Country (reference)
(n)			(kcals)	(g/d^c^ or g/kg BW^d^)	(g/d^c^ or g/kg BW^d^)	(g/d^c^ or g/kg BW^d^)	(software)	(derived estimate or instrument)	
12 (21)	29.2 ± 6.3	58.1 ± 6.9	2008 ± 431	284.4 ± 91.4^c^	66.5 ± 16.6^c^	63.3 ± 27.7^c^	3 d food diary (FoodProcessor; ESHA)	NR	USA (8)
20 (22)		54.8 ± 5.0	1621 ± 453	266.1 ± 67.9^c^	61.1 ± 21.1^c^	37.2 ± 23.0^c^			
NR (25)	22.4 ± 3.2^a^	64.9 ± 8.9^a^	1878 ± 543	241.0 ± 79.0^c^	77.0 ± 24.0^c^	NR	3 d food recall (software not reported)	NR	USA (6)
19 ± 1 (10)	NR	55.1 ± 5.1	1734 ± 328	47% energy 3.66 ± 0.67^d^	1.34 ± 0.17^d^	35.3% energy 1.23 ± 0.29^d^	7 d food diary (Polish Institute of Food & Nutrition Tables)	NR	Poland (11)
9-16 (22)	25.4 ± 4.0	56.3 ± 6.5	1882 ± 403	255.0 ± 59.0^c^	72.0 ± 17.0^c^	73.0 ± 21.0^c^	3 d food diary (FoodProcessor; ESHA)	Estimated BMR (Tanita BCA 545)	Poland (12)
17-29 (14)	25.4 ± 3.5	58.2 ± 5.3	1953 ± 403	271.0 ± 59.0^c^	74.0 ± 17.0^c^	70.0 ± 21.0^c^			
19.2 ± 3.1 (9)	13.3 ± 2.2	46.6 ± 8.5	2047 ± 649	3.9 ± 1.5^d^	1.6 ± 0.3^d^	66.0 ± 17.0^c^	3 d food recall (Nutritics)	Estimated RMR (Nutritics; Schofield)	USA (13)
21.1 (20)	31.4 ± 7.7	58.5 ± 5.7	2039 ± 267	220.5 ± 46.3^c^	92.6 ± 29.6^c^	84.0 ± 18.9^c^	3 d weighed food (Nutritics)	NR	UK (14)
14.5 ± 2.3 (11)	34.9 ± 8.5	56.4 ± 4.1	*1897* ± *578*	*40.1% energy* *190.6* ^c^	*15.8% energy* *74.4* ^c^	*38.9% energy* *81.9* ^c^	3 d food diary (DIAL® Alce Ingeniería)	Estimated BMR (WHO) Estimated TEE (PA diary, METs)	Spain (15)
16.7 ± 23.9 (26)	30.1 ± 7.1	58.5 ± 6.3	1902 ± 373	226.8 ± 60.3^c^ 3.92 ± 1.06^d^	80.2 ± 16.5^c^ 1.39 ± 0.34^d^	65.7 ± 14.2^c^ 1.13 ± 0.24^d^	3 d food diary (FoodProcessor; ESHA)	Measured RMR (indirect calorimetry; Cosmed Fitmate)	Poland (16)
19.9 ± 2.3 (14)	15.9 ± 1.5	51.7 ± 4.3	1710 ± 460	*232.2* *±* *11.1*^c^ *4.3* *±* *1.3*^a^^,^^d^	*70.2* *±* *0.5*^c^ *1.3* *±* *0.4*^a^^,^^d^	32 ± 5% energy^a^ *63.2* *±* *2.4*^a,c^	3 d weighed food (OPEN)	Estimated EEE (PA diary, METs)	Slovenia (17)
16.3 ± 3.5 (40)	30.1 ± 7.0	57.4 ± 6.2	1917 ± 399	226.6 ± 59.7^c^ 4.41^d^	79.5 ± 16.2^c^ 1.36^d^	68.1 ± 19.4^c^ 1.57^d^	3 d food diary (Food Processor; ESHA)	Measured RMR (indirect calorimetry; Cosmed Fitmate) Estimated EEE (questionnaire & PA diary, METs)	Poland (18)
20.3 ± 2.4 (11)	27.6 ± 6.9	57.09 ± 7.02	1775 ± 351	192.1 ± 44.7^c^ 3.4 ± 0.7^d^	72.2 ± 30.0^c^ 1.3 ± 0.5^d^	77.2 ± 20.3^c^ 1.4 ± 0.4^d^	4 d food diary (Nutritics)	Estimated RMR (Cunningham) Estimated EEE (accelerometry, HR, & PA diary)	Ireland (19)
18.2 ± 4.7 (23)	28.0 ± 8.0	55.7 ± 5.5	1447 ± 313	13.05 ± 44.7	73.6 ± 18.4	65.9 ± 15.8	3 d food diary (DietPro)	Estimated EEE (questionnaire, METs)	Spain (20)
NR (17)	26.9 ± 4.2	52.4 ± 8.0	1335 (446)^d^	144.0 (48.8)^c^ 2.5 (1.3)^d^	54.2 (17.0)^c^ 1.0 (0.3)^d^	62.7 ± 21.7^c^ 1.1 (0.7)^d^	7 d diet history multi-pass method (Nutritionist Pro)	Measured REE (indirect calorimetry; Cortex MetaMax) Estimated TEE (questionnaire & PA recall, METs)	Malaysia (21)

Reported dietary data assumed from food and beverage only, excluding dietary supplements, in female subjects (*n* = 285) with at least 6 months of climbing experience. Absolute energy and macronutrients are presented as mean ± SD unless otherwise noted.

n, Number of subjects; y, year; kg, kilogram; kcal, kilocalorie; g/d, grams per day; BW, body weight; NR, not reported; BMR, basal metabolic rate; RMR, resting metabolic rate; REE, resting energy expenditure; PA, physical activity; TEE, total energy expenditure; EEE, exercise energy expenditure; HR, heart rate; METs, metabolic equivalents. *Italic font:* estimated from reported data.

a Averaged with male data in same study.

b Data reported as median (interquartile).

c g/d.

d g/kg BW.

### Energy and macronutrients

Thirteen published articles specifically collected dietary intake data on individuals that climb on rock and artificial surfaces (9–21). Most studies reported males with a higher average energy intake compared to females. Adult female energy intake ranged from 1,335 ± 446 to 2,047 ± 649 kilocalories (kcals). The lower end is especially concerning, as this is likely not adequate to support most females' basal metabolic needs. Adult male energy intake ranged from 1,633 ± 528 to 3,556 ± 477 kcals, but these were exceptions, as most males averaged between 2,100–2,700 kcals daily. In addition, these studies found that absolute carbohydrate, protein, and fat intake were also higher in males than females, likely due to the overall increased energy intake. In these studies, males had a larger body mass average than females, and thus on average, most likely had higher energy and macronutrient requirements.

Between 28%–88% of participants in the studies with targeted dietary energy and/or macronutrient standards did not meet them. Five studies (14, 17–20) specifically assessed for low energy availability, and of these, between 36%–88% of the participants presented with suboptimal energy availability [<45 kcal/kg/fat-free mass (FFM)/day] and 28%–59% had low energy availability (<30 kcal/kg FFM/day).

In studies examining nutrition and climbing abilities (13, 16, 18, 20), Przeliorz-Pyszczek et al. (12) was the only study to find the beginner/intermediate group had lower energy, carbohydrate, protein, and fat intake compared to the advanced/elite group. The other studies found no significant differences between climbing abilities and energy or macronutrient intake.

Compared with other similar sport athletes, a review on indoor sport team athletes found most athletes did not meet energy and carbohydrate targets (23). Eighty-five percent of those athletes were below the recommended range of 6 grams per kilogram of bodyweight (g/kg BW)/day. While sports included volleyball, basketball, handball, and futsal with movement patterns different than climbers, some commonalities exist, including training time, intermittent anaerobic metabolism, and the impact of repeated falls. Castillo et al. (23) also found adequate intake of protein in most athletes, which is consistent with much of the dietary intake data for climbers thus far.

Gymnasts also perform moves with gravitational aspects related to some climbing movements. In a study on male collegiate gymnasts, Kuhlman et al. (24) reported similar results with 85.7% of their athletes in a state of low energy availability, due to insufficient energy and carbohydrate intakes.

Dietary protein consumption varied across studies from 72 to 126 g for male climbers with deviations as large as 50 g and females consumed 54–93 g with deviations as big as 30 g, but most achieved targets set by the researchers. Among the analyzed diets, with the exception of the study by En et al. (21), protein intake generally fell within the recommended range for athletes of 1.2–2 g/kg BW (2). However, not all studies stratified their participants according to climbing discipline, ability, or training/exercise hours, factors that may influence daily protein needs.

The proportion of dietary energy from fat, according to recommendations of 20%–35% of total energy intake (2), was met in the majority of participants in most studies, yet three studies found average intakes exceeded this standard by about 5% (15, 19, 20). In studies where dietary fat quality was analyzed, the intake of saturated fatty acids (SFA) exceeded recommendations of 10% of total energy intake (12, 15, 18, 20). Overconsumption of fat was also observed in the review with indoor athletes by Castillo et al. (23). Additionally, a similar pattern of high total fat and SFA intake was reported among high-performance Lithuanian athletes from various sports disciplines, where reducing SFA intake was considered a necessary step to enhance the anti-inflammatory potential of the diet (25).

Of notable mention, specific climbing groups may be particularly prone to general macronutrient insufficiencies when reporting overall low dietary energy (17, 20, 21) or may fluctuate in those with severe budget constraints climbing in wilderness locations (10).

### Micronutrients

Among the eleven published studies assessing selected micronutrients in their climber participants (9, 10, 12, 14–21), most reported an average underconsumption of target vitamin and/or mineral standard values per country-based recommendations. The eight studies that assessed dietary iron in females found insufficient values, in both adults and adolescents (12, 14, 15, 17–21). Vitamin D intakes were also very low in all seven published studies that examined this nutrient in both females and males (12, 15, 17–21). Notably, when reported dietary supplement use was included in the diet analysis, micronutrient averages were markedly higher and only two studies mentioned this (14, 20), suggesting failure to account for dietary supplement ingestion may lead to inaccurate estimations of micronutrient intake in study participants.

Since nutrient deficiencies may be driven by low dietary intakes, climbers may be susceptible to nutrient deficiencies and their health outcomes. The prevalence of iron deficiency among the general female athlete population has been reported as high as 60% (26), identifying this group as particularly vulnerable. Vitamin D deficiency has been reported in 30% of elite athletes and 36% of adolescents (27), compared with a global prevalence of 15.7% (28). Although micronutrient intake should be interpreted in the context of population-specific recommendations, which vary by country and change over time, several studies in this minireview identified insufficient dietary intakes of other micronutrients besides iron and vitamin D.

Concerning dietary intake levels of iodine (12, 15, 18, 20), zinc (15), folate (15), potassium (15, 18), magnesium (19), and calcium (15, 18, 19) were found. Interestingly, Chmielewska et al. (18) demonstrated that elite female climbers exhibited the highest overall diet quality among the analyzed performance levels, suggesting that advanced climbing ability may be associated with greater nutritional awareness and more micronutrient-dense dietary patterns. However, very few participants had elite climbing status and very few studies have stratified their participants by ability, thus future research will help to determine if elite climbers are more likely to eat higher amounts of micronutrients compared with novice climbers. Additionally, no research has determined specific amounts of micronutrients climbers require although it seems likely that micronutrient guidelines established for healthy populations and for general athletes would be similar.

### Fluids

Only three studies reported fluid intakes of their participants, although none measured hydration status. In the study by Przeliorz-Pyszczek et al. (12), 3-day average fluid intakes were 2,486 ± 703 mL in the female beginner/intermediate (IRCRA 9–16) group and males consumed 3,053 ± 1,225 mL. In the same study, the advanced/elite (IRCRA 17–29) female climbers consumed 2,887 ± 725 mL of fluid and males consumed 3,020 ± 922 mL on average over 3 days. The researchers noted that regardless of their climbing ability, their diets included more than 2,000 mL of liquids a day. Further, Kemmler et al. (9), recorded 2,861 ± 861 mL of water per day on average over 5 days in fifteen elite-high elite (IRCRA 28–26) male climbers. Last, Merrells et al. (10) followed two young males on a low-budget outdoor climb trip using a diary and food scale to weigh and record food/beverage for 29 days with hydration maintained with an average daily water intake of 2,000–3,000 mL. These three studies represented 118 climbing athletes that roughly consumed between 2,000–4,000 mL of fluids daily. Clearly, more data is needed to discern appropriateness of fluid intake for climbers, competitive or not.

### Timing/periodization/temporal aspects

Important aspects of sports nutrition include the timing of fuel (i.e., pre-training carbohydrates, post-training protein) surrounding training on a given day as well as periodized nutrition throughout a season to match training periodization. These aspects of dietary intake have been suggested to enhance training which may lead to enhanced athletic performance (29–31). Rest days may require less total dietary energy compared to training days, depending on restoration aspects of the climber.

Few researchers attempted to discern some dietary differences between climb days and rest days. Úbeda et al. (15) had climbers record their food over three days (no climbing, indoor gym climbing, outdoor natural rock climbing), and found statistical differences for dietary energy averages between days, but did not report when eating occurred in relation to the day's climbing. En et al. (21), also reported dietary data for training days and non-training days over seven days. They found their male and female climbers ate about 50% of their estimated energy expended on non-training day and less than 50% on training days, but also did not report when food was eaten relative to when climbing occurred on training days.

In four studies, participants were described as competitive or reported to have competed in climbing events (11, 13, 17, 20). Little attention was given to the timing of nutrition in these athletes. For example, Sas-Nowosielski et al. (11) stated their participants were “members of an athletic section in one climbing gym in Katowice” and that their dietary data was recorded for seven days “during their usual training and climbing activity within a preparation period.” However, details about when food was consumed within a given day were not provided. Simič et al. (17) recruited participants from the Slovenia Youth Climbing team during the national team selection camp and assessed dietary intake from three consecutive days, including one rest day. They neglected to mention when or which types of foods were consumed before, during or after the training took place on the two training days or report energy on training days compared to the rest day. Further, Mora-Fernandez et al. (20) collected 3 days of diet data during climbing camps on non-consecutive days, but also did not state when daily training took place with respect to when food was consumed.

More studies are needed to help support competitive climbing athletes in this space. Expanding the number of days to collect dietary data within a particular training phase as well as when foods are consumed throughout a given day with respect for when training sessions occur within the same day will help to elucidate whether nutrition is appropriate to support training for competitive climbers' and ultimately their performances. No studies have collected dietary data during climbing competitions, thus another sports nutrition area to examine in future observational research.

## Discussion

The purpose of this minireview was to critique the methodology shortcomings of dietary data collected in observational studies. Results unveiled sixteen observational diet studies including 658 climbers (43% female; ages 10–42y) in different disciplines with varied experience, only some competitive. From a scientific perspective, the quality of these observations was concerning.

Determining the total caloric demand of climbing athletes is a methodological challenge. Total energy expenditure (TEE) is often described as three components: basal energy expenditure, diet-induced thermogenesis, and physical activity thermogenesis. Each of these components can be influenced by many factors, including age, sex, genetics, body composition, and diet type (32).

Resting metabolic rate (RMR) refers to the energy necessary to sustain essential physiological processes at rest, including substrate metabolism, respiration, thermoregulation, and cardiac function (33). Indirect calorimetry has been regarded as a decent method for RMR assessment (34); however, its high cost and labor-intensive nature restrict its routine application in clinical settings. Only three studies used this method to assess either RMR or resting energy expenditure (REE) (16, 18, 21).

In contrast, movement energy is more difficult to objectively quantify. Thus, energy requirements of climbers are typically estimated using predictive equations designed for other athletic populations, which may result in discrepancies between predicted and actual RMR values (35). The doubly labeled water (DLW) method may be more appropriate to help ascertain TEE over longer durations (weeks), with support wearing accelerometers and heart rate monitors. The DLW method is considered the gold standard for measuring TEE due to its high accuracy, noninvasiveness, and minimal burden on participants, who can maintain normal activities during the measurement period (36). Albeit, none of the existing studies to date have used this method to determine the energy expenditure of climbing athletes. Instead, most studies in this minireview that assessed energy expenditure calculated Physical Activity Level (PAL) or Metabolic Equivalent of Task (MET) values based on self-reported diaries and declared training hours. Although these methods place little burden on participants, they rely on memory of past activities, increasing the risk of recall bias (36). Given the widespread application of dietary assessment methods in sports nutrition, concerns remain since the validity of these methodologies were not subjected to sufficiently rigorous evaluation within this specific population (37).

Discrepancies between self-reported dietary intake and estimated energy expenditure have been consistently documented in athletes of different ages, sexes, and sporting disciplines. Retrospective self-reporting methods, such as food records, dietary recalls, and diet histories, are particularly susceptible to error due to deliberate or unintentional underreporting, as well as changes in habitual dietary practices during the assessment period (37).

While there is no universally accepted gold standard for measuring dietary energy intake, the food record remains the most commonly used method in sports nutrition research and practice (38). The weighed food record approach requires participants to document the weights of all food and beverage consumed over a specified period, placing considerable demands on their accuracy and honesty, and on researchers to code the data correctly by navigating appropriate food/nutrient databases. In athletic populations, these limitations are further exacerbated by the high volume of food intake, numerous and sometimes irregular eating occasions, challenges in estimating large portion sizes, contribution of non-database foods such as sports-specific products (i.e., energy bars, gels, sports drinks), and dietary supplements (39).

In summary, the amassed data suggests many climbers don't meet germane nutrition standards indicating vulnerability to nutrient inadequacies if food practices remain consistent. However, meager methodology may be liable. These studies assessed very few days of food intake, which may not reflect food behavior during different phases of training over longer periods of time. The data was collected via convenient sampling with heterogenous climbing abilities represented, few of which were known competitors with a variety of different nutrient assessment methods. Furthermore, future publications need to clarify dietary supplement contributions to daily intake to assess the necessity/efficacy of supplements. Energy needs may be better determined using enhanced techniques, such as DLW over longer periodized training phases simultaneously with different weeks of 7-d weighed food records over a competitive season. Ideally, objective biometric analyses (i.e., blood/urine analyses) would add to reported dietary intakes for enhanced determination of nutrient status in these athletes.

The competitive climbing population requires further investigation, including elite or high-elite abilities (IRCRA), different climbing disciplines, adolescents, and geographically diverse cohorts. Thus, to determine how sports nutrition may be optimized to support international competitive climbing athletes' training regimes, further investigation is required with attention to these details. Until published evidence reveals these important aspects, the best sports nutrition for climbing athletes is a sports dietitian's best guess.
