# Supplementary material for: Nutrition prescription for climbing athletes on rock or artificial surfaces: are we guessing?
Source: Front Sports Act Living. 2026 Jul 31;8:1799533. doi: 10.3389/fspor.2026.1799533 (PMC13474986; doi:10.3389/fspor.2026.1799533)
Supplement: Supplementary file 1 [file Supplementaryfile1.pdf]

Supplementary Material Table S1 Database Search Terms.
Databases and respective search-terms with their Boolean techniques.

Database	Terms
PubMed	(climb*[MeSH] OR Boulder* OR speed) AND (eat* OR nutrients OR diet* [Mesh] OR food) AND (athlete* OR youth OR adolescent*) NOT stairs NOT plant* NOT ladder
Scholar	Limited to articles:<br>[nutrients of human climbers] -stairs -plants -ladder -rodents -fish -mountain
CINAHL Plus with full text	(climb* or sport climbing or indoor climbing or bouldering) AND (nutrition or diet or food or nourishment or eat) AND human NOT stairs
ScienceDirect	(climb* OR Boulder* OR speed) AND (eat* OR nutrients OR diet* OR food) AND (athlete* OR youth OR adolescent*) NOT stairs NOT plant* NOT ladder
