# Supplementary material for: Nutrition prescription for climbing athletes on rock or artificial surfaces: are we guessing?
Source: Front Sports Act Living. 2026 Jul 31;8:1799533. doi: 10.3389/fspor.2026.1799533 (PMC13474986; doi:10.3389/fspor.2026.1799533)
Supplement: Supplementary file 2 [file Supplementaryfile2.pdf]

Supplement Material Table S2 Male Micronutrients.

Reported dietary data assumed from food and beverage only, excluding dietary supplements, in male subjects (n=312) with at least 6 months of climbing experience. Micronutrient data presented as mean±SD unless otherwise noted. Symbols: [*averaged with female data in same study], [^data reported as median (interquartile)], [**data reported as % Spanish Dietary Recommended Intake]; Abbreviations: reference (Ref.), not reported (NR), milligrams (mg), micrograms (μ), vitamin (vit); calcium (ca); iron (Fe), sodium (Na), potassium (K), phosphorus (P), magnesium (Mg), zinc (Zn), copper (Cu), manganese (Mn), selenium (Se), iodine (I), International Rock Climbing Research Association (IRCRA).

	Ref. 6 (n=15)	Ref. 10 (n=2)	Ref. 12 (n=65)	Ref. 14 (n=20)	Ref. 15 (n=34)**	Ref. 16 (n=29)	Ref. 17 (n=13)	Ref. 18 (n=66)	Ref. 19 (n=14)	Ref. 20 (n=22)	Ref. 21 (n=32)^
**Vit A μ/d**	NR	514.3± 578.4	1537.7± 1369.0	NR	158.3± 77.4	NR	NR	1512.7± 1876.1	NR	868.0± 714.3	248.6 (400.4)
**Vit B1 mg/d**	NR	NR	1.6± 0.7	NR	137.1± 73.7	NR	NR	1.5± 1.0	1.6± 0.7	1.53± 0.51	0.4 (0.4)
**Vit B2 mg/d**	1.8± 0.9	NR	2.6± 1.2	NR	112.9± 60.3	NR	NR	2.2± 1.2	NR	2.1± 1.1	0.6 (0.8)
**Vit B3 mg/d**	20.9± 11.6	NR	NR	NR	245.4± 228.8	NR	NR	22.0± 10.7	33.5± 17.9	33.9± 15.0	5.9 (6.8)
**Vit B6 mg/d**	NR	NR	2.7± 1.1	NR	148.7± 44.6	NR	NR	2.5± 1.3	NR	2.4± 0.8	22.1 (50.4)
**Folate μ/d**	NR	NR	445.1± 191.0	NR	78.7± 27.5	NR	NR	462.5± 207.2	NR	333.2± 129.9	0.3 (0.5)
**Vit B12 μ/d**	NR	NR	6.6± 4.3	NR	290.4± 130.4	NR	NR	5.5± 6.7	1.5± 3.9	20.5± 60.1	0.3 (0.5
**Vit C mg/d**	139.2± 80.3	NR	137.1± 86.1	NR	248.4± 135.1	155.0± 86.0	NR	145.4± 95.3	77.1± 54.4	146.1± 101.2	28.3 (37.1)
**Vit D μ/d**	3.5± 3.8	NR	4.5± 3.9	NR	23.4± 16.5	NR	2.6± 2.3	4.3± 3.7	3.6± 2.6	12.8± 24.9	0.0 (0.4)
**Vit E mg/d**	8.2± 64.0	NR	14.8± 6.8	NR	84.8± 37.1	13.0± 0.6	NR	13.5± 6.6	NR	15.2± 6.1	1.8 (2.7))
**Ca mg/d**	NR	NR	1268.8± 534.6	NR	80.4± 21.7	NR	780.0± 300.0	928.5± 335.4	682.0± 459.0	949.1± 371.2	310.0 (379.9)
**Fe mg/d**	16.2± 9.1	NR	17.5± 6.4	14.1± 7.7	149.9± 46.9	NR	14.0± 5.0	15.8± 4.6	10.5	16.3± 5.6	6.4 (7.2)
**Na mg/d**	2658.5± 684.0	NR	2821.5± 1256.4	NR	NR	NR	NR	2357.6± 920.5	NR	NR	NR
**K mg/d**	2467.3± 910.7	NR	4228.2± 1253.5	NR	85.0± 22.9	NR	NR	3479.9± 985.8	NR	3496.6± 1253.1	579.4 (498.3
**P mg/d**	NR	NR	1996.0± 704.1	NR	206.6± 51.8	NR	NR	1610.7± 444.8	NR	1454.23± 392.27	354.6 (318.2)
**Mg mg/d**	NR	NR	511.5± 186.7	NR	93.1± 28.7	NR	NR	404.6± 125.5	318± 163	413.7± 193.0	48.8 (40.6)
**Zn mg/d**	NR	NR	14.6± 5.5	NR	64.7± 20.7	NR	NR	12.3± 3.5	NR	11.0± 3.0	1.5 (1.6)
**Cu μ/d**	NR	NR	2.0± 0.9	NR	NR	NR	NR	1.8± 0.6	NR	NR	NR
**Mn mg/d**	NR	NR	7.7± 5.2	NR	NR	NR	NR	5.8± 3.3	NR	NR	NR
**Se μ/d**	NR	NR	NR	NR	158.6± 49.1	NR	NR	20.5± 41.1	NR	83.9± 47.7	3.5 (12.4)
**I μ/d**	NR	NR	65.8± 47.8	NR	65.8± 25.8	NR	NR	43.0± 2.9	NR	127.8± 151.4	0.0 (4.8)
**Energy kcal**	2125± 472	3556± 477	2543± 566	2270±5 62	2123± 648	2217± 429	1880± 470	2260± 508	2480± 572	2177± 505	1633 (528)
**Weight kg**	64.9± 8.9*	82.5	71.1± 6.2	72.4± 7.5	69.4± 6.9	73.0± 7.9	56.4± 11.0	72.4± 7.7	69.7± 8.2	67.8± 7.7	65.0± 10.0
**Age y**	22.4± 3.2*	21	26.2± 4.8	27.8± 6.1	34.3± 8.0	30.2± 5.7	15.5± 1.6	30.9± 6.1	26.2± 6.4	32± 10.0	27.8± 4.7
**Climb Ability IRCRA**	NR	14.5	9-16	17-29	19.1± 3.9	16.9± 3.3	21.2± 2.2	17.7± 4.1	23.0± 3.4	20.0± 4.6	NR
