# Supplementary material for: Nutrition prescription for climbing athletes on rock or artificial surfaces: are we guessing?
Source: Front Sports Act Living. 2026 Jul 31;8:1799533. doi: 10.3389/fspor.2026.1799533 (PMC13474986; doi:10.3389/fspor.2026.1799533)
Supplement: Supplementary file 3 [file Supplementaryfile3.pdf]

Supplement Material Table S3 Females Micronutrients.

Reported dietary data assumed from food and beverage only, excluding dietary supplements, in female subjects (n=223) with at least 6 months of climbing experience. Micronutrient data presented as mean±SD unless otherwise noted. Symbols: [*averaged with male data in same study], [^data reported as median (interquartile)], [**data reported as % Spanish Dietary Recommended Intake]; Abbreviations: reference (Ref.), not reported (NR), milligrams (mg), micrograms (μ), vitamin (vit); calcium (ca); iron (Fe), sodium (Na), potassium (K), phosphorus (P), magnesium (Mg), zinc (Zn), copper (Cu), manganese (Mn), selenium (Se), iodine (I), International Rock Climbing Research Association (IRCRA).

	Ref. 6 (n=25)	Ref. 12 (n=36)	Ref. 15 (n=11)**	Ref. 14 (n=20)	Ref. 16 (n=26)	Ref. 17 (n=14)	Ref. 18 (n=40)	Ref. 19 (n=11)	Ref. 20 (n=23)	Ref. 21 (n=17)^
**Vit A μ/d**	NR	1490.3± 857.7	168.4± 84.2	NR	NR	NR	1430.4± 912.9	NR	880.5± 405.3	151.2 (214.2)
**Vit B1 mg/d**	NR	1.35± 0.47	101.2± 44.8	NR	NR	NR	1.3± 0.7	1.0	1.2± 0.7	0.2 (0.2)
**Vit B2 mg/d**	NR	1.80± 1.32	98.2± 29.5	NR	NR	NR	1.7± 0.6	NR	1.6± 1.1	0.3 (0.4)
**Vit B3 mg/d**	NR	NR	305.5± 261.2	NR	NR	NR	18.2± 7.1	25.8± 14.2	114.9± 165.3	3.0 (3.3)
**Vit B6 mg/d**	NR	2.15± 0.70	119.5± 22.5	NR	NR	NR	2.2± 0.8	NR	1.9± 0.9	0.2 (0.3)
**Folate μ/d**	NR	372.3± 122.8	63.7± 19.6	NR	NR	NR	415.2± 139.2	NR	311.2± 157.2	25.5 (41.8)
**Vit B12 μ/d**	NR	3.55± 2.32	248.9± 116.4	NR	NR	NR	4.1± 3.1	3.7± 2.6	34.0± 83.1	0.1 (0.3)
**Vit C mg/d**	NR	158.1± 78.2	241.0± 117.2	NR	155± 82	NR	169.0± 77.3	113.7± 97.8	189.8± 244.9	28.2 (43.9)
**Vit D μ/d**	3.8± 5.0	3.60± 2.70	11.8± 7.0	NR	NR	2.6± 2.3	4.4± 3.9	3.7± 3.1	8.0± 10.5	0.0 (0.5)
**Vit E mg/d**	5.7± 4.0	11.05± 3.65	72.2± 27.2	NR	13± 0.62	NR	12.0± 5.6	NR	23.2± 54.3	0.6 (1.8)
**Ca mg/d**	NR	898.4± 315.6	83.3± 29.2	NR	NR	780± 300	877,05± 830.2	529± 205	788.2± 328.4	151.9 (250.2)
**Fe mg/d**	NR	13.8± 4.2	71.7± 21.5	13.4± 3.8	NR	10± 5	14.6± 3.8	7.6± 3.1	12.9± 6.5	3.0 (2.8)
**Na mg/d**	2689.1± 933.9	1671.0± 760.4	NR	NR	NR	NR	2172.3± 844.0	NR	NR	NR
**K mg/d**	1992.2± 803.1	3664.3± 935.8	73.7± 13.5	NR	NR	NR	3248.8± 791.5	NR	2323.6± 818.8	388.7 (242.3)
**P mg/d**	NR	1336.2± 311.9	191.5± 59.6	NR	NR	NR	1321.7± 262.0	NR	1008.5± 304.7	210.2 (193.7)
**Mg mg/d**	219.7± 16.4	400.0± 95.9	91.0± 19.8	NR	NR	NR	392.9± 220.9	229±68	288.5± 124.8	22.5 (37.6)
**Zn mg/d**	NR	10.1± 2.4	59.3± 20.4	NR	NR	NR	10.2± 2.3	NR	9.9± 12.9	0.6 (0.5)
**Cu μ/d**	NR	1.80± 0.50	NR	NR	NR	NR	1.75± 0.60	NR	NR	NR
**Mn mg/d**	NR	6.1± 2.3	NR	NR	NR	NR	4.9± 2.7	NR	NR	NR
**Se μ/d**	NR	NR	180.3± 49.4	NR	NR	NR	17.0± 27.7	NR	40.0± 16.1	0.2 (8.4)
**I μ/d**	NR	44.1± 17.4	74.1± 16.8	NR	NR	NR	43.9± 38.4	NR	68.4± 48.7	0.0 (2.3)
**Energy kcal**	1878± 543	1910± 403	1897± 578	2039± 26	1902± 373	1710± 460	1917± 399	1775± 351	1447± 313	1335 (446)
**Weight kg**	64.9± 8.9*	57.0± 6.0	56.4± 4.1	58.5± 5.7	58.5± 6.3	51.7± 4.3	57.4± 6.2	57.1± 7.0	52.0± 4.7	52.4± 8.0
**Age y**	22.4± 3.2*	25.4± 3.8	34.9± 8.5	31.4± 7.7	30.1± 7.1	15.9± 1.5	30.1± 7.0	27.6± 6.9	28.0± 8.0	26.9± 4.2
**Climb Ability IRCRA**	NR	9-16 17-29	14.5± 2.3	21.1	16.7± 23.9	15.9± 1.5	16.3± 3.5	20.3± 2.4	18.2± 4.7	NR
